# Differential roles of the prefrontal cortical subregions and basolateral amygdala in compulsive cocaine seeking and relapse after voluntary abstinence in rats

**DOI:** 10.1111/ejn.12289

**Published:** 2013-07-01

**Authors:** Yann Pelloux, Jennifer E Murray, Barry J Everitt

**Affiliations:** 1 Institut de Neuroscience de la Timone, UMR 7289 CNRS, Université d'Aix-Marseille27, Bld Jean Moulin, 13385, Marseille, France; 2 Department of Psychology, University of CambridgeDowning St, CB2 3EB, Cambridge, UK; 3 Behavioural and Clinical Neuroscience Institute, University of CambridgeDowning St, CB2 3EB, Cambridge, UK

**Keywords:** cocaine self-administration, compulsivity, prefontal cortex, relapse, top-down inhibitory control

## Abstract

Compulsive drug use and a persistent vulnerability to relapse are key features of addiction. Imaging studies have suggested that these features may result from deficits in prefrontal cortical structure and function, and thereby impaired top-down inhibitory control over limbic–striatal mechanisms of drug-seeking behaviour. We tested the hypothesis that selective damage to distinct subregions of the prefrontal cortex, or to the amygdala, after a short history of cocaine taking would: (i) result in compulsive cocaine seeking at a time when it would not usually be displayed; or (ii) facilitate relapse to drug seeking after abstinence. Rats with selective, bilateral excitotoxic lesions of the basolateral amygdala or anterior cingulate, prelimbic, infralimbic, orbitofrontal or anterior insular cortices were trained to self-administer cocaine under a seeking–taking chained schedule. Intermittent mild footshock punishment of the cocaine-seeking response was then introduced. No prefrontal cortical lesion affected the ability of rats to withhold their seeking responses. However, rats with lesions to the basolateral amygdala increased their cocaine-seeking responses under punishment and were impaired in their acquisition of conditioned fear. Following a 7-day abstinence period, rats were re-exposed to the drug-seeking environment for assessment of relapse in the absence of punishment or cocaine. Rats with prelimbic cortex lesions showed decreased seeking responses during relapse, whereas those with anterior insular cortex lesions showed an increase. Combined, these results show that acute impairment of prefrontal cortical function does not result in compulsive cocaine seeking after a short history of self-administering cocaine, but further implicates subregions of the prefrontal cortex in relapse.

## Introduction

Compulsive drug use and its resumption after abstinence in addicts occur even in the face of conflict with other incentives, including fear of punishment and the loss of alternative sources of reinforcement (Waldorf *et al*., 1991). This has been modelled in rats with procedures whereby, in order for them to self-administer cocaine, seeking responses must be made despite the threat of (Vanderschuren & Everitt, 2004) or actual punishment of seeking (Pelloux *et al*., 2007, 2012) or taking (Deroche-Gamonet *et al*., 2004) responses. Previous studies investigating punishment-induced suppression of cocaine seeking or taking have demonstrated that, after a long, but not a short, cocaine-taking history, ∼20% of animals persist in compulsively seeking cocaine (Deroche-Gamonet *et al*., 2004; Pelloux *et al*., 2007, 2012), thereby providing an opportunity to investigate the neural basis of this vulnerability.

Increasing evidence has suggested that poor inhibitory control resulting from sub-optimal functioning of prefrontal cortical structures (Volkow & Fowler, 2000; Naqvi & Bechara, 2009; Ersche *et al*., 2012) that may precede (Volkow & Fowler, 2000; Ersche *et al*., 2012), or be the result of, chronic exposure to addictive drugs may provide an important mechanism underlying the propensity to lose control over drug intake and to seek cocaine compulsively (Volkow *et al*., 1992; Grant *et al*., 1996; Rogers *et al*., 1999; Hester & Garavan, 2004; Ersche *et al*., 2005). Individuals addicted to cocaine or other drugs of abuse show decreased grey matter volume in several subregions of the prefrontal cortex (PFC), including the orbitofrontal cortex (OFC), anterior cingulate cortex (ACC), and insular cortex (IC) (Franklin *et al*., 2002; Matochik *et al*., 2003; Ersche *et al*., 2011, 2012), as well as alterations in the amygdala, albeit with less consistent findings (Makris *et al*., 2004; Ersche *et al*., 2012), and white matter changes suggesting disrupted connectivity between cortical and striatal structures (Ersche *et al*., 2012). Preclinical animal models have demonstrated a progressive reduction in prefrontal cortical metabolic activity over the course of a long cocaine self-administration history (Macey *et al*., 2004; Porrino *et al*., 2007), and rats treated with or having self-administered cocaine show perseveration in reversal learning (Schoenbaum *et al*., 2004; Calu *et al*., 2007) that mirrors that seen in humans addicted to cocaine (Ersche *et al*., 2005), indicating impaired OFC function, as perseverative or compulsive responding is seen in humans with lesions of the OFC (Tsuchida *et al*., 2010), as well as in non-human primates (Dias *et al*., 1996). Moreover pre-training (Schoenbaum *et al*., 2003) lesions of the OFC in rats result in perseverative responding, a form of compulsive behaviour, in a reversal learning task. Taken together, these data suggest that compulsive cocaine use may reflect PFC dysfunction that could pre-date exposure to the drug and hence predispose to its emergence as a consequence of repeated drug exposure.

Relapse to cocaine seeking, measured as the reinstatement of responding following extinction of the instrumental taking response, has also been shown to depend, in part, on the PFC and basolateral amygdala (BLA) (Kalivas & McFarland, 2003; Peters *et al*., 2008, 2009), and an associated dysregulation of glutamate transmission in the nucleus accumbens (Kalivas & Volkow, 2011). Inactivation of the BLA or the medial PFC prevents instrumental extinction from suppressing cocaine-seeking behaviour. The propensity to relapse is also predicted by trait impulsivity (Economidou *et al*., 2009; Broos *et al*., 2012), and impulsivity is itself markedly increased by pre-training (Mobini *et al*., 2002) or post-training lesions of the PFC (Chudasama *et al*., 2003) or the BLA (Winstanley *et al*., 2004). Therefore, impulse control deficits are associated with the propensity to seek cocaine compulsively (Belin *et al*., 2008) and the propensity to relapse after abstinence.

We have tested the hypothesis that pre-training impairments of prefrontal cortical areas or the amygdala, that have been shown to result in impulsive and compulsive behaviours, would increases the likelihood of compulsive cocaine seeking, measured as resistance to intermittent punishment of seeking responses, at a time after a brief cocaine self-administration history when this behaviour has not emerged in the general population of rats (Pelloux *et al*., 2007, 2012). We also assessed the resumption of drug seeking during re-exposure to the drug-seeking context after abstinence, which has been shown previously to be exacerbated in impulsive rats (Economidou *et al*., 2009) and to be influenced by medial prefrontal, especially infralimbic, cortical mechanisms (Peters *et al*., 2009).

## Materials and methods

### Subjects

Male outbred Lister hooded rats (Charles River, UK), weighing ∼250 g upon arrival, were housed in pairs in polycarbonate cages (length, 40 cm; width, 25 cm; height, 18 cm), and maintained under a reversed 12-h light/dark cycle (lights on at 19:00 h) at a constant temperature (21 ± 1 °C), with free access to laboratory chow (SDS) and water. Following surgery, rats were housed individually, and fed 20 g of food each day within 1 h of testing. Water was always freely available in the home cage. The experimental procedures were conducted in accordance with the United Kingdom 1986 Animals (Scientific Procedures) Act (project licence PPL 80/2234).

### Surgery

The rats were anaesthetized with Avertin [2% (w/v) 2,2,2-tribromoethanol, 1% (w/v) 2-methylbutan-2-ol and 8% (v/v) ethanol in phosphate-buffered saline, 10 mL/kg, intraperitoneal]. All rats were implanted with a catheter (CamCaths, Cambridge, UK) in the right jugular vein targeting the left vena cava. The mesh end of the catheter was sutured subcutaneously on the dorsum between the scapulae. Rats were then placed in a stereotaxic frame (David Kopf Instruments, Tujunga, CA, USA) with the incisor bar set at 3.3 mm below the interaural line (Paxinos & Watson, 1998). Axon-sparing excitotoxic lesions were made by infusing 0.09 m quinolinic acid (Sigma, Poole, UK) in 0.1 m phosphate buffer (pH 7.2–7.4) via a 30-gauge stainless steel cannula connected by fine-bore polythene tubing (internal diameter, 0.28 mm; internal diameter, 0.16 mm; Portex, UK) to a Hamilton precision microsyringe mounted in an infusion pump (Harvard Apparatus, UK). The stereotaxic coordinates for each targeted area are shown in Table S1. All dorsal–ventral coordinates were taken from dura, and anterior–posterior coordinates were measured from bregma. Each infusion of 0.4–0.6 μL, depending on the structure targeted (Table S1), was made over 3 min, and the cannulae were left in place for a further 3 min. Rats receiving sham surgery were injected identically with phosphate buffer vehicle.

Throughout 1 week of recovery, rats were treated daily with 10 mg/kg subcutaneous Baytril (Genus Express, Bury St Edmunds, UK) to prevent infection (Caine *et al*., 1992).

### Apparatus

Cocaine self-administration training and testing took place in 12 operant conditioning chambers (29.5 × 32.5 × 23.5 cm; Med Associates, Georgia, VT, USA) equipped with two 4-cm-wide retractable levers that were mounted in one sidewall 12 cm apart and 8 cm from the grid floor. Above each lever there was a cue light (2.5 W, 24 V), and a house light (2.5 W, 24 V) was located on the opposite wall. A dipper delivered 0.04 mL of a 20% (w/v) sucrose solution to a recessed magazine (3.8 cm high and 3.8 cm wide and 5.5 cm from the grid floor) situated between the levers. Entry into this magazine was detected by the interruption of an infrared photo-beam. The floor of the chamber was covered with a metal grid with bars separated by 1 cm and connected to a shock generator and scrambler (Med Associates), which delivered 0.5-mA footshocks. The grid was located 8 cm above an empty tray. The testing chamber was placed within a sound-attenuating and light-attenuating enclosure equipped with a ventilation fan that also screened external noise. Silastic tubing shielded with a metal spring extended from each rat's intravenous catheter to a liquid swivel (Stoelting, Wood Dale, IL, USA) mounted on an arm fixed outside the operant conditioning chamber. Tygon tubing extended from the swivel to a Razel infusion pump (Semat Technical, UK) located outside the outer enclosure. The operant conditioning chambers were controlled by software written in C++ with the Whisker control system (Cardinal & Aitken, 2010). Fear conditioning took place in four specially configured operant chambers (Paul Fray, Cambridge, UK), which were controlled by a computer through basic software. The house light and three lights on the front wall were illuminated during conditioning, and a loudspeaker was mounted on the ceiling. The floor of the chamber was covered with a metal grid with bars separated by 1 cm and connected to a shock generator and scrambler (Campden Instruments, UK), which delivered 0.5-mA footshocks. A video camera was mounted on the ceiling and connected to a DVD recorder for later scoring of freezing behaviour.

### Procedure

**Figure 1:**
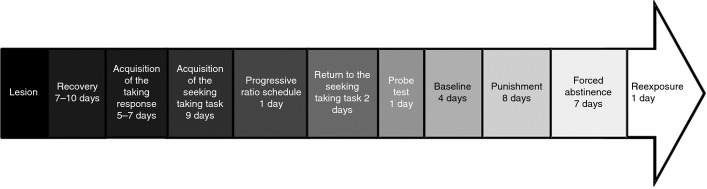
Flowchart of the simplified experimental procedure. For details, see Materials and methods.

The timeline of the procedure is shown in Fig. 1.

### Acquisition of the taking response

Behavioural training began 7–10 days after surgery. Each session began with the insertion of the taking lever (left–right counterbalanced across rats in each group). Responding was reinforced under a fixed ratio (FR) 1 schedule. Each lever press produced a 0.25-mg cocaine infusion (0.1 mL/5 s) accompanied by the withdrawal of the taking lever, the extinction of the house light, and the illumination of the stimulus light above the lever for 20 s. The sessions terminated after 30 cocaine infusions or 2 h, depending on which criterion was met first. Training of the taking response continued for five to seven sessions.

### Training of the seeking–taking chained schedule of cocaine reinforcement

Following acquisition of the taking schedule, the seeking–taking chain schedule was introduced, each cycle beginning with insertion of the drug-seeking lever with the taking lever retracted; the first press on the seeking lever initiated a random interval (RI) schedule on the seeking lever. The RI parameter was progressively increased from 2 to 120 s. The first lever press after the RI had elapsed terminated the first link of the chain, resulting in the retraction of the seeking lever and insertion of the taking lever to initiate the second link. One press on the taking lever was followed by the drug infusion accompanied by the same stimulus events as during the training of the taking response. There was then a time-out (TO) period, which was progressively increased across sessions from 20 s to 10 min after each cocaine infusion. The seeking lever was then reinserted to start the next ‘cycle’ of the schedule. Consequently, at the end of five training sessions, the rats were responding on a heterogeneous chained (tandem FR 1, RI 120 s) FR 1 TO schedule, allowing a maximum of 11 cocaine infusions.

### Motivational measures: progressive ratio; seeking responses under extinction test, and baseline seeking response rate

To investigate whether the incentive value of cocaine was affected by lesions of the different PFC subregions or the BLA, we assessed responding on the taking lever under a progressive ratio schedule. The ratio requirement of the taking response was increased after each reinforcer according to the following progression: 1, 3, 6, 9, 12, 17, 24, 32, 42, 56, 73, 95, 124, 161, 208, 268, 346, 445, 573, and 737 (Richardson & Roberts, 1996). The value of the last ratio completed was taken as the break point. The session ended either after 4 h or after 40 min without an earned infusion.

Following progressive ratio assessment, rats were returned to the seeking–taking chain for 2 days, after which performance of the seeking response was measured in the absence of the taking lever and cocaine infusions (Olmstead *et al*., 2000).

All groups then received a further three sessions on the seeking–taking chain, in which rats were also trained to nose poke into the magazine for sucrose (0.04 mL of a 20% solution) delivered under a VI schedule, which progressively increased (from 2 and 15 s) to 60 s on the third day. This last day was considered to be the first day of baseline. Responding for sucrose allowed for the assessment of general suppression of appetitive behaviour by, and thus the specificity of the effects of, the subsequent introduction of intermittent punishment of the cocaine-seeking response.

Three further sessions of training under the seeking–taking chain with concurrent sucrose reinforcement established a baseline in which performance during the seeking link of the chain is monotonically related to the cocaine dose under the TO conditions of the session (Olmstead *et al*., 2000), thereby providing an additional measure of the motivational impact of the different cortical and amygdala lesions.

### Punishment

Finally, the introduction of intermittent punishment upon completion of the seeking link of the schedule was introduced over eight consecutive days (Pelloux *et al*., 2007). During each punishment session, a randomly selected half of the cycles contained no punishment and were reinforced identically to those in baseline training. In the remaining cycles, completion of the seeking response component was punished, the talking lever was not presented, and no cocaine was delivered (Pelloux *et al*., 2007). The first response that met the RI requirement in the seeking link delivered the 0.5-s foot shock and led to a direct transition to the TO period without the taking link. Consequently, individual punishment sessions consisted of a maximum of five to six non-punished cycles in which rats had the opportunity to self-administer cocaine, interspersed with five to six punished cycles with no cocaine-taking opportunity.

### Abstinence and reinstatement

After the last day of assessment of cocaine seeking under punishment, all rats were left undisturbed in their home cages for 7 days without access to cocaine. On the day following this period of enforced abstinence, the several groups of PFC-lesioned rats and their corresponding sham controls were re-exposed to the operant chamber for a non-reinforced session under the seeking–taking chained schedule; that is, all reinforcers, including punishment, were omitted (Economidou *et al*., 2009). BLA-lesioned rats were not tested, as they showed no evidence of punishment-induced abstinence (see Results).

One week after the last day of punishment, BLA-lesioned rats and the respective controls underwent Pavlovian fear conditioning.

### Conditioned fear

Two minutes following introduction into a fear-conditioning chamber (Paul Fray), a clicker was presented for 1 min, which ended with the delivery of a single footshock (2 s, 0.5 mA). One minute later, the rat was returned to its home cage. After 24 h, the rat was returned to the conditioning chamber for a 20-min test session, during which the clicker was alternately switched on (1 min) and off (1 min). Freezing was scored every 5 s during the test session.

### Histological assessment

At the end of the experiment, rats were deeply anaesthetized with intraperitoneal Euthatal (pentobarbitone sodium, 200 mg/mL; 1.5 mL) and perfused transcardially with 0.01 m phosphate-buffered saline followed by 4% (w/v) paraformaldehyde in phosphate-buffered saline. Their brains were removed and postfixed in paraformaldehyde before being dehydrated in 20% (w/v) sucrose; they were then sectioned coronally at a thickness of 60 μm on a freezing microtome, and every third section was mounted and allowed to dry. Sections were passed through a series of ethanol solutions of descending concentration [3 min in each of 100, 95 and 70% (v/v) ethanol in water], and stained for 5 min with Cresyl Violet [0.05% (w/v) aqueous Cresyl Violet, 2 mm acetic acid and 5 mm formic acid in water]. After staining, sections were rinsed in water and 70% ethanol before being differentiated in 95% ethanol. Finally, they were dehydrated and delipidified in 100% ethanol and Histoclear (National Diagnostics, Hessle, UK) before being cover-slipped and allowed to dry. The sections were used to verify lesion placement and assess the extent of quinolinate-induced neuronal loss and gliosis.

### Statistical analyses

Between-subjects anovas were conducted for the progressive ratio, extinction probe test, and baseline seeking–taking motivational measures. Seeking under punishment was analysed with a mixed two-way anova with group as the between-subjects measure and session as the within-subject measure. Seeking at reinstatement was assessed with a between-groups one-way anova. Significance was set at *α* = 0.05. Significant interactions were analysed further with protected Fisher's LSD *post hoc* analyses.

## Results

### Motivational measures

**Figure 2:**
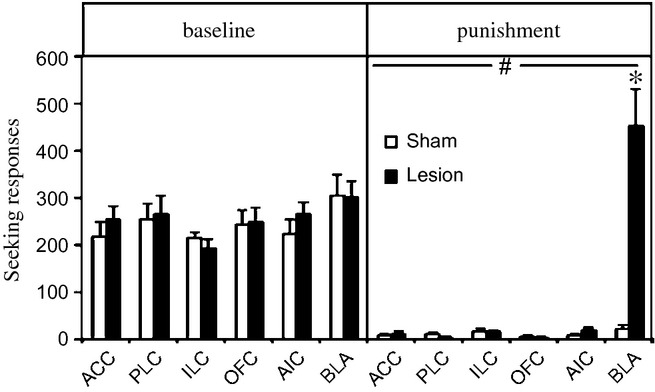
Average number of seeking responses during the 4 days before (baseline; left side) or during the last 4 days (punishment; right side) of punishment of rats with lesions to the ACC, PLC, ILC, OFC, AIC and BLA (black bars) and their counterpart sham controls (white bars). Data are expressed as mean ± standard error of the mean of 10–16 rats per group. Significantly different from baseline: ^#^*P* < 0.001. Significant difference between sham and lesioned rats: *P < 0.001.

After training on the seeking–taking task, and before introduction of the punishment contingency, the effects of the PFC and BLA lesions on the motivation to respond for cocaine were measured. The multiple analysis of variance performed on the break points under the progressive ratio schedule (Fig. S1; top panel), on seeking performance during the non-reinforced seeking probe test (Fig. S1; bottom panel) or during baseline responding (Fig. 2; left panel) revealed no significant effect of any lesion on any motivational measure (treatment × region interaction: *F*_5,133_ < 2.1).

### Seeking under intermittent punishment

All rats with ACC, prelimbic cortex (PLC), infralimbic cortex (ILC), OFC or anterior IC (AIC) prefrontal lesions suppressed their seeking responses to the same extent as controls, i.e. abstained from responding. In contrast, rats with lesions to the BLA both maintained (group × phase interaction: *F*_5,133_ = 12.4; *P* < 0.001) and actually increased their cocaine-seeking responses during the punishment sessions, thereby showing evidence of compulsive behaviour (*P* < 0.001) (Fig. 2; right panel). Within individual punishment sessions, sham-operated rats showed a pronounced increase in latency to seeking after punishment (Fisher's LSD, *P* < 0.001), whereas BLA-lesioned rats showed increased responding (*P* < 0.05) after both cocaine and shock as compared with that seen during the first cycle, and thus no sign of suppression across the session (Fig. S4).

The majority of rats showed an initial decrease in concomitant responding for sucrose following introduction of the punishment contingency (ACC, *F*_11,220_ = 11; PLC, *F*_11,209_ = 4.7; OFC, *F*_11,231_ = 8.7; AIC, *F*_11,297_ = 9) for 2–7 days (*P* < 0.05), but this soon returned to baseline. However, BLA-lesioned and ILC-lesioned rats differed from their respective sham controls (BLA, *F*_11,220_ = 2; ILC, *F*_11,231_ = 2.9) by maintaining stable responding for sucrose across baseline and the early phase of the intermittent punishment contingency (Fig. S2).

### Reinstatement of cocaine seeking after abstinence

**Figure 3:**
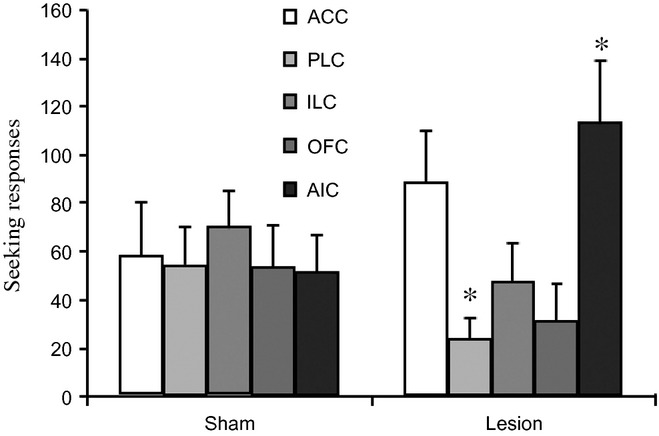
Number of seeking responses during re-exposure in rats with lesions to the ACC (white histogram), PLC (light grey), ILC (grey), OFC (dark grey) and AIC (light black) (right side) and their counterpart sham controls (left side). Data are expressed as mean ± standard error of the mean of 10–16 rats per group. Significant difference between sham and lesioned rats: *P < 0.05.

Prefrontal cortical lesions differentially affected the reinstatement of drug-seeking responses after the return of rats to the test environment (*F*_4,113_ = 3.5; *P* = 0.01) (Fig. 3). AIC-lesioned rats showed increased seeking responses as compared with sham controls (*P* = 0.02), and there was a similar trend for ACC-lesioned rats (*P* = 0.16). In contrast, PLC-lesioned rats decreased their seeking responses (*P* = 0.03), similar to the trend observed in rats with OFC or ILC lesions (*P* > 0.23).

### Assessment of fear conditioning in rats with BLA lesions

Given the marked increase in cocaine seeking under punishment in the BLA-lesioned rats, they and their respective sham controls were subjected to fear conditioning to assess the extent to which reduction in fear evoked by shock was a factor in the persistent seeking of cocaine. BLA-lesioned rats showed a reduction in conditioned fear, as expressed by a significant decrease in freezing in response to the conditioned stimulus previously associated with footshock as compared with sham controls (Student's *t*-test: *t*_1,19_ = −2.6, *P* = 0.02) (Fig. S3).

### Histological assessment

**Figure 4:**
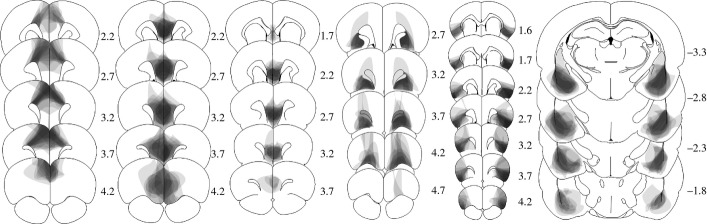
Schematic representations of the lesions on standardized sections of the rat brain. From left to right: ACC, PLC, ILC, OFC, AIC, and BLA. The numbers adjoining each section refer to distances from bregma (adapted from Paxinos & Watson, 1998).

Histological assessment was performed after completion of the experiment, and was conducted blind to the behavioural results. The sites and extent of lesions are illustrated in Fig. 4. Lesioned rats included in the behavioural analysis sustained lesions to the majority of the structure of interest, with minimal involvement of adjacent structures. Relative to bregma, neuronal loss and associated gliosis extended from approximately 1.7–4.5 mm in the ACC, 1.7–4.7 mm in the PLC, 1.7–3.7 mm in the ILC, 2.2–4.7 mm in the OFC, 1.0–4.7 mm in the AIC, and −1.5 to −4.5 mm in the BLA.

## Discussion

In this study, we tested the hypothesis that prefrontal cortical or limbic cortical loss of function plays a causal role in the development of compulsive cocaine seeking, as suggested by correlations between reduced grey matter volume or reduced metabolic activity and addiction to cocaine in human populations (Volkow *et al*., 1992; Franklin *et al*., 2002; Matochik *et al*., 2003; Ersche *et al*., 2011, 2012). We have previously shown that a significant proportion of rats with a long, but not a short, history of cocaine self-administration seek cocaine compulsively, in that they persist in making seeking responses despite intermittent and unpredictable punishment of the seeking response (Pelloux *et al*., 2007, 2012). In the present experiments, we investigated whether irreversible loss of function in prefrontal or limbic cortical areas would result in an increased propensity to seek cocaine compulsively in rats with a short cocaine-taking history, at a stage when none display this behaviour (Deroche-Gamonet *et al*., 2004; Vanderschuren & Everitt, 2004; Pelloux *et al*., 2007; Belin *et al*., 2008). Withholding of cocaine-seeking responses under unpredictable punishment also provides a model of abstinence that does not depend on the prior extinction of cocaine-taking responses, but upon the revaluation of the actions required to obtain the drug under threat of punishment. We further investigated the impact of these selective lesions of several prefrontal cortical areas on the tendency of rats to relapse after abstinence.

The results showed that pre-cocaine exposure lesions of the ACC, PLC, ILC, OFC or AIC did not result in compulsive cocaine seeking, as measured by its resistance to punishment. Lesions of the BLA, however, resulted in both resistance to punishment-induced suppression and an increase in cocaine seeking, but the characteristics of this apparent compulsive cocaine seeking were somewhat different from that seen in the subpopulation of intact rats in which this behaviour has emerged over a long cocaine self-administration history. By contrast, there was evidence of opposing influences of the AIC and PLC on the reinstatement of cocaine seeking after abstinence; rats with AIC lesions showed an enhanced propensity to relapse, whereas those with PLC lesions showed a reduced propensity to relapse. The results are consistent with the notion of top-down inhibitory control by the PFC of maladaptive drug-seeking behaviour during relapse.

Lesions of the IC are known to reduce aversive (Bermudez-Rattoni & McGaugh, 1991) or frustrative non-reward-motivated (Kesner & Gilbert, 2007) behaviour and to favour risky decision-making in humans (Clark *et al*., 2008). However, patients who have sustained IC lesions because of stroke were shown to be less prone to resume smoking than abstinent patients with lesions elsewhere in the brain (Naqvi *et al*., 2007). This apparent discrepancy between clinical observations and the effects of acute manipulations studied here could reflect the precise location of lesions in the context of the heterogeneous structure of the IC. In rats, the posterior IC is associated with cocaine craving (Contreras *et al*., 2007) but not with avoidance learning (Dunn & Everitt, 1988), whereas the anterior region that was targeted in the present study is necessary for the acquisition and retrieval of avoidance memories (Bermudez-Rattoni *et al*., 1997). Lesions of the AIC did not influence any measure of the motivation for cocaine, including break points under a progressive ratio schedule of reinforcement, baseline seeking rates, or seeking during probe tests. The push–pull mechanisms that determine cocaine-seeking performance under a punishment contingency might therefore engage different parts of the IC from those which, when damaged in humans, result in a decreased tendency to relapse.

Lesions of the PLC, in contrast, decreased the propensity to relapse when abstinent rats were returned to the drug-seeking environment. These results therefore indicate opposing influences of the PLC and AIC on relapse following punishment-induced abstinence. Opposing effects of ILC and ACC lesions on relapse following extinction have also been demonstrated, with functional inactivation of the ILC favouring, and inactivation of the ACC preventing, reinstatement (Peters *et al*., 2008, 2009; LaLumiere *et al*., 2010). Thus, the reinstatement of cocaine seeking following abstinence achieved either through the threat of aversive consequences or the extinction of instrumental seeking responses depends upon dissociable prefrontal networks, the activity of which mediates both the suppression and performance of seeking responses.

The lack of effect of PFC lesions on the tendency to maintain or even increase cocaine seeking under intermittent punishment conditions is perhaps surprising, particularly in the case of OFC lesions. In rats, pre-training lesions of the PFC similar to those used here result in perseverative responding, a form of compulsive behaviour, in a reversal learning task (Schoenbaum *et al*., 2003), and in impulsivity in a serial reaction-time task (Mobini *et al*., 2002). Impulsivity is a trait that predisposes to the development of compulsive cocaine seeking (Belin *et al*., 2008). In rats self-administering cocaine, lesions of the OFC and medial PFC, including the ACC, resulted in an increased tendency to respond instrumentally for cocaine (Weissenborn *et al*., 1997; Hutcheson & Everitt, 2003), but, perhaps importantly, these changes were not seen under conditions in which there was a risk of punishment. In a structural imaging study of cocaine-dependent individuals, greater compulsivity of cocaine use was associated with reduced volume of the OFC, and the drug-dependent subjects, in general, showed reduced grey matter volumes in the cingulate, insular and temporo-parietal cortices (Ersche *et al*., 2011). Cocaine-dependent individuals also showed reduced baseline activity of the OFC (Volkow *et al*., 1992) and reduced activation of the ACC during a go/no go task, and this correlated with difficulty in inhibiting the pre-potent response (Kaufman *et al*., 2003; Hester & Garavan, 2004). Individuals addicted to stimulants show reduced neurocognitive function (Goldstein *et al*., 2004), as well as risky behaviour and poor decision-making in gambling tasks that are similar to the effects of OFC or IC lesions (Rogers *et al*., 1999; Clark *et al*., 2008). These individuals also showed compulsive responding in a reversal learning task (Ersche *et al.,* 2008).

The lack of effect of the various prefrontal lesions on compulsive cocaine seeking clearly demonstrates that acute loss of function of the PFC is not, in itself, sufficient to result in compulsive cocaine seeking. This could reflect the fact that acute, pre-cocaine self-administration lesions do not capture the subtle nature of either pre-existing reductions in grey matter volume reflecting a vulnerable endophenotype (Ersche *et al*., 2012), or cocaine-induced alterations in PFC structure and function that are only apparent after a prolonged self-administration history (Porrino *et al*., 2007). Structural and functional changes in cocaine-dependent subjects are not restricted to cortical structures, but also include the striatum and the amygdala. The putamen has a significantly greater volume in both cocaine-dependent individuals and their non-drug-dependent siblings, suggesting that this may be a pre-existing or vulnerability factor that might be related to a greater propensity for habitual drug use (Ersche *et al*., 2012). Consistent with this view is our finding that reversible inactivation of the dorsolateral striatum, which is homologous with the putamen in the primate brain and has been shown to underlie habitual cocaine seeking (Zapata *et al*., 2010), reduces cocaine seeking selectively under punishment conditions (Jonkman *et al*., 2012).

In addition, decreased dopaminergic and serotoninergic activity in the PFC has been reported in drug-addicted individuals (Wilson, 1996) and in monkeys with a prolonged self-administration history (Porrino *et al*., 2007). Moreover, the 20% of rats that seek cocaine compulsively after a long history of cocaine intake also show a marked reduction of serotonin utilization in prefrontal cortical areas, as well as in limbic cortical and striatal structures, as compared with non-compulsive rats, despite the same history of cocaine exposure (Pelloux *et al*., 2012). Depleting the forebrain of serotonin in rats with a short history of cocaine intake – similar to the stage at which rats were studied in the present experiments – did result in an increased tendency to seek cocaine under punishment (Pelloux *et al*., 2012). Reducing the levels of serotonin in the OFC of monkeys (modelling a deficit seen in cocaine-addicted individuals) (Wilson, 1996) results in perseverative responding during reversal learning (Clarke *et al*., 2004). Such reduced serotoninergic activity may be associated with disinhibited PFC neuronal activity (Puig & Gulledge, 2011). Notably, the OFC hypoactivity seen at rest in addicted humans contrasts with its hyperactivity during the presentation of drug-associated cues (Childress *et al*., 1999; Volkow & Fowler, 2000). Pre-cocaine experience lesions of the PFC precluded the investigation of this phenomenon in the present experiments, so this phenomenon could not be investigated further. Assessment of the impact on compulsive cocaine seeking of serotonergic impairment or enhancement in the PFC may be illuminating, but the present data clearly refute the hypothesis that compulsive cocaine seeking results from acutely impaired PFC function.

Using identical behavioural procedures to those used here, we have previously shown that the noradrenaline reuptake inhibitor atomoxetine also differentially affects responding under intermittent, unpredictable punishment and responding during relapse. Whereas acute systemic atomoxetine administration prevented relapse after voluntary abstinence (Economidou *et al*., 2009), it did not affect cocaine seeking under punishment (Pelloux *et al*., 2012). Therefore, it is likely that the propensity to relapse and the maintenance of responding for cocaine despite adverse consequences, two key features of drug addiction, may engage dissociable neurochemical mechanisms.

Rats with lesions of the BLA not only continued to respond despite the punishment contingency, but actually showed a marked increase in cocaine seeking above the pre-punishment baseline. This increase is unlikely to be attributable to baseline drift, as performance stabilized across the four baseline sessions, and previous data have clearly shown that non-punished animals do not increase their performance over time (Pelloux *et al*., 2007). The failure of BLA-lesioned animals to suppress behaviour under the threat of shock may best be attributed to their inability to form a fear memory, because they also show a reduction in conditioned fear, as has been demonstrated in many studies (Hitchcock & Davis, 1986; Dunn & Everitt, 1988; LeDoux *et al*., 1990; Killcross *et al*., 1997). Recent studies using an identical task to that established by Pelloux *et al*. (2007) and employed here showed that post-training inactivation of the central nucleus of the amygdala (CeN) also reduced the effect of punishment on cocaine seeking (Xue *et al*., 2012). However, the results of the two studies differ somewhat; in the present study, BLA lesions not only prevented the reduction in seeking performance following punishment, but actually increased cocaine seeking, whereas CeN inactivation selectively prevented punishment-induced suppression. Paradoxically, non-contingent presentation of footshock is able to reinstate previously extinguished cocaine seeking, an effect that requires the integrity of the CeN (McFarland *et al*., 2004). Therefore, BLA-lesioned rats with an intact CeN may remain sensitive to the arousing effect of shock, and hence increase their baseline seeking rate, but are unable to suppress their seeking behaviour in the face of intermittent punishment, owing to an inability to associate the contingency between seeking responses and punishment, consistent with the demonstration of a double dissociation in the effects of BLA and CeN lesions on punishment and Pavlovian conditioned suppression, respectively, on appetitive responding (Killcross *et al*., 1997).

However, although these data suggest that the persistence of cocaine seeking under punishment may reflect amygdala dysfunction, a general loss of fear does not provide an explanation for the compulsive cocaine seeking that emerges over a long period of cocaine self-administration, as we have shown that this can occur without any deficit in fear conditioning (Vanderschuren & Everitt, 2004; Pelloux *et al*., 2007, 2012). Thus, although acute lesions of the BLA (herein) or inactivation of the CeN (Xue *et al*., 2012) result in increased cocaine seeking through a loss of fear, the compulsive cocaine seeking in a vulnerable 20% of animals over a long period of cocaine exposure emerges progressively without any change in fear processing (Pelloux *et al*., 2007, 2012). This dissociation indicates that causal explanations for compulsive cocaine seeking require the identification of neural, perhaps neurochemical (Pelloux *et al*., 2012), adaptations that are not captured by acute reversible or reversible inactivations of the amygdala or other neural loci.

In conclusion, the results of these experiments show that lesions of the BLA, but not of prefrontal cortical areas, can markedly increase the propensity to seek cocaine under punishment after a short cocaine-taking history, but that this effect does not capture the mechanisms underlying the emergence of compulsive cocaine seeking after a prolonged or escalated cocaine-taking history. Prefrontal cortical lesions that were without effect on compulsive cocaine seeking nevertheless influenced relapse after abstinence, with PLC lesions preventing and AIC lesions increasing relapse. These findings further indicate inhibitory control mechanisms that are dissociable from those underlying relapse in extinction–reinstatement models of relapse behaviour.

## Abbreviations

ACC: anterior cingulate cortex
AIC: anterior insular cortex
BLA: basolateral amygdala
CeN: central nucleus of the amygdala
FR: fixed ratio
IC: insular cortex
ILC: infralimbic cortex
OFC: orbitofrontal cortex
PFC: prefrontal cortex
PLC: prelimbic cortex
RI: random interval
TO: time-out

## Supporting Information

Fig S1 Break point under the progressive ratio schedule and seeking responses during the non-reinforced seeking probe test in which only the seeking lever was presented in rats with lesions to the anterior cingulate cortex, prelimbic cortex, infralimbic cortex, orbitofrontal cortex, anterior insular cortex and basolateral amygdala and their counterpart sham controls.

Fig S2 Number of nose poke responses over the 4 days before (baseline) or during the 8 days (punishment) of punishment of rats with lesions to the anterior cingulate cortex, prelimbic cortex, infralimbic cortex, orbitofrontal cortex, anterior insular cortex and basolateral amygdala and their counterpart sham controls.

Fig S3 Percentage of time spent in freezing during intermittent presentation of a 1-min conditioned stimuli in rats with lesions to the basolateral amygdala and their counterpart sham controls.

Fig S4 Latency to the first seeking response and seeking rate during baseline or during punishment either during the first cycle, or after a previous cocaine-reinforced cycle, or after a previously punished cycle, in rats with lesions to the BLA basolateral amygdala and their counterpart sham controls.

Table S1 Infusion coordinates for infusion (Chudasama et al., 2003; Ito et al., 2005; Boulougouris et al., 2007; Kesner & Gilbert, 2007).

## References

[b1] Belin D, Mar AC, Dalley JW, Robbins TW, Everitt BJ (2008). High impulsivity predicts the switch to compulsive cocaine-taking. Science.

[b2] Bermudez-Rattoni F, McGaugh JL (1991). Insular cortex and amygdala lesions differentially affect acquisition on inhibitory avoidance and conditioned taste aversion. Brain Res.

[b3] Bermudez-Rattoni F, Introini-Collison I, Coleman-Mesches K, McGaugh JL (1997). Insular cortex and amygdala lesions induced after aversive training impair retention: effects of degree of training. Neurobiol. Learn. Mem.

[b5] Broos N, Diergaarde L, Schoffelmeer AN, Pattij T, Vries De TJ (2012). Trait impulsive choice predicts resistance to extinction and propensity to relapse to cocaine seeking: a bidirectional investigation. Neuropsychopharmacol.

[b6] Caine SB, Lintz R, Koob GF, Sahgal A (1992). Intravenous self-administration techniques in animals. Behavioral Neuroscience: A Practical Approach.

[b7] Calu DJ, Stalnaker TA, Franz TM, Singh T, Shaham Y, Schoenbaum G (2007). Withdrawal from cocaine self-administration produces long-lasting deficits in orbitofrontal-dependent reversal learning in rats. Learn. Memory.

[b8] Cardinal RN, Aitken MR (2010). Whisker: a client-server high-performance multimedia research control system. Behav. Res. Methods.

[b9] Childress AR, Mozley PD, McElgin W, Fitzgerald J, Reivich M, O'Brien CP (1999). Limbic activation during cue-induced cocaine craving. Am. J. Psychiat.

[b10] Chudasama Y, Passetti F, Rhodes SE, Lopian D, Desai A, Robbins TW (2003). Dissociable aspects of performance on the 5-choice serial reaction time task following lesions of the dorsal anterior cingulate, infralimbic and orbitofrontal cortex in the rat: differential effects on selectivity, impulsivity and compulsivity. Behav. Brain Res.

[b11] Clark L, Bechara A, Damasio H, Aitken MR, Sahakian BJ, Robbins TW (2008). Differential effects of insular and ventromedial prefrontal cortex lesions on risky decision-making. Brain.

[b12] Clarke HF, Dalley JW, Crofts HS, Robbins TW, Roberts AC (2004). Cognitive inflexibility after prefrontal serotonin depletion. Science.

[b13] Contreras M, Ceric F, Torrealba F (2007). Inactivation of the interoceptive insula disrupts drug craving and malaise induced by lithium. Science.

[b14] Deroche-Gamonet V, Belin D, Piazza P (2004). Evidence for addiction-like behavior in the rat. Science.

[b15] Dias R, Robbins TW, Roberts AC (1996). Primate analogue of the Wisconsin Cart Sorting Test: effects of excitotoxic lesions of the prefrontal cortex in the marmoset. Behav. Neurosci.

[b16] Dunn LT, Everitt BJ (1988). Double dissociations of the effects of amygdala and insular cortex lesions on conditioned taste aversion, passive avoidance, and neophobia in the rat using the excitotoxin ibotenic acid. Behav. Neurosci.

[b17] Economidou D, Pelloux Y, Robbins TW, Dalley JW, Everitt BJ (2009). High impulsivity predicts relapse to cocaine-seeking after punishment-induced abstinence. Biol. Psychiat.

[b18] Ersche KD, Fletcher PC, Lewis SJ, Clark L, Stocks-Gee G, London M, Deakin JB, Robbins TW, Sahakian BJ (2005). Abnormal frontal activations related to decision-making in current and former amphetamine and opiate dependent individuals. Psychopharmacology.

[b504] Ersche KD, Roiser JP, Robbins TW, Sahakian BJ (2008). Chronic cocaine but not chronic amphetamine use is associated with perseverative responding in humans. Psychopharmacology.

[b19] Ersche KD, Barnes A, Jones PS, Morein-Zamir S, Robbins TW, Bullmore ET (2011). Abnormal structure of frontostriatal brain systems is associated with aspects of impulsivity and compulsivity in cocaine dependence. Brain.

[b20] Ersche KD, Jones PS, Williams GB, Turton AJ, Robbins TW, Bullmore ET (2012). Abnormal brain structure implicated in stimulant drug addiction. Science.

[b21] Franklin TR, Acton PD, Maldjian JA, Gray JD, Croft JR, Dackis CA, O'Brien CP, Childress AR (2002). Decreased gray matter concentration in the insular, orbitofrontal, cingulate, and temporal cortices of cocaine patients. Biol. Psychiat.

[b22] Goldstein RZ, Leskovjan AC, Hoff AL, Hitzemann R, Bashan F, Khalsa SS, Wang GJ, Fowler JS, Volkow ND (2004). Severity of neuropsychological impairment in cocaine and alcohol addiction: association with metabolism in the prefrontal cortex. Neuropsychologia.

[b23] Grant S, London ED, Newlin DB, Villemagne VL, Liu X, Contoreggi C, Phillips RL, Kimes AS, Margolin A (1996). Activation of memory circuits during cue-elicited cocaine craving. Proc. Natl. Acad. Sci. USA.

[b24] Hester R, Garavan H (2004). Executive dysfunction in cocaine addiction: evidence for discordant frontal, cingulate, and cerebellar activity. J. Neurosci.

[b25] Hitchcock J, Davis M (1986). Lesions of the amygdala, but not of the cerebellum or red nucleus, block conditioned fear as measured with the potentiated startle paradigm. Behav. Neurosci.

[b26] Hutcheson DM, Everitt BJ (2003). The effects of selective orbitofrontal cortex lesions on the acquisition and performance of cue-controlled cocaine seeking in rats. Ann. NY Acad. Sci.

[b28] Jonkman S, Pelloux Y, Everitt BJ (2012). Differential roles of the dorsolateral and midlateral striatum in punished cocaine seeking. J. Neurosci.

[b29] Kalivas PW, McFarland K (2003). Brain circuitry and the reinstatement of cocaine-seeking behavior. Psychopharmacology.

[b30] Kalivas PW, Volkow ND (2011). New medications for drug addiction hiding in glutamatergic neuroplasticity. Mol. Psychiatr.

[b31] Kaufman JN, Ross TJ, Stein EA, Garavan H (2003). Cingulate hypoactivity in cocaine users during a GO-NOGO task as revealed by event-related functional magnetic resonance imaging. J. Neurosci.

[b32] Kesner RP, Gilbert PE (2007). The role of the agranular insular cortex in anticipation of reward contrast. Neurobiol. Learn. Mem.

[b33] Killcross S, Robbins TW, Everitt BJ (1997). Different types of fear conditioned behaviour mediated by separate nuclei within amygdala. Nature.

[b34] LaLumiere RT, Niehoff KE, Kalivas PW (2010). The infralimbic cortex regulates the consolidation of extinction after cocaine self-administration. Learn. Memory.

[b35] LeDoux JE, Cicchetti P, Xagoraris A, Romanski L-M (1990). The lateral amygdaloid nucleus: sensory interface of the amygdala in fear conditioning. J. Neurosci.

[b36] Macey DJ, Rice WN, Freedland CS, Whitlow CT, Porrino LJ (2004). Patterns of functional activity associated with cocaine self-administration in the rat change over time. Psychopharmacology.

[b37] Makris N, Gasic GP, Seidman LJ, Goldstein JM, Gastfriend DR, Elman I, Albaugh MD, Hodge SM, Ziegler DA, Sheahan FS, Caviness VS, Tsuang MT, Kennedy DN, Hyman SE, Rosen BR, Breiter HC (2004). Decreased absolute amygdala volume in cocaine addicts. Neuron.

[b38] Matochik JA, London ED, Eldreth DA, Cadet JL, Bolla KI (2003). Frontal cortical tissue composition in abstinent cocaine abusers: a magnetic resonance imaging study. NeuroImage.

[b39] McFarland K, Davidge SB, Lapish CC, Kalivas PW (2004). Limbic and motor circuitry underlying footshock-induced reinstatement of cocaine-seeking behavior. J. Neurosci.

[b200] Mobini S, Body S, Ho MY, Bradshaw CM, Szabadi E, Deakin JF, Anderson IM (2002). Effects of lesions of the orbitofrontal cortex on sensitivity to delayed and probabilistic reinforcement. Psychopharmacology.

[b40] Naqvi NH, Bechara A (2009). The hidden island of addiction: the insula. Trends Neurosci.

[b41] Naqvi NH, Rudrauf D, Damasio H, Bechara A (2007). Damage to the insula disrupts addiction to cigarette smoking. Science.

[b42] Olmstead MC, Parkinson JA, Miles FJ, Everitt BJ, Dickinson A (2000). Cocaine-seeking by rats: regulation, reinforcement and activation. Psychopharmacology.

[b43] Paxinos G, Watson C (1998). The Rat Brain in Stereotaxic Coordinates.

[b44] Pelloux Y, Everitt BJ, Dickinson A (2007). Compulsive drug seeking by rats under punishment: effects of drug taking history. Psychopharmacology.

[b45] Pelloux Y, Dilleen R, Economidou D, Theobald D, Everitt BJ (2012). Reduced forebrain serotonin transmission is causally involved in the development of compulsive cocaine seeking in rats. Neuropsychopharmacol.

[b46] Peters J, LaLumiere RT, Kalivas PW (2008). Infralimbic prefrontal cortex is responsible for inhibiting cocaine seeking in extinguished rats. J. Neurosci.

[b47] Peters J, Kalivas PW, Quirk GJ (2009). Extinction circuits for fear and addiction overlap in prefrontal cortex. Learn. Memory.

[b48] Porrino LJ, Smith HR, Nader MA, Beveridge TJ (2007). The effects of cocaine: a shifting target over the course of addiction. Prog. Neuro.-Psychoph.

[b49] Puig MV, Gulledge AT (2011). Serotonin and prefrontal cortex function: neurons, networks, and circuits. Mol. Neurobiol.

[b50] Richardson NR, Roberts DC (1996). Progressive ratio schedules in drug self-administration studies in rats: a method to evaluate reinforcing efficacy. J. Neurosci. Meth.

[b51] Rogers RD, Everitt BJ, Baldacchino A, Blackshaw AJ, Swainson R, Wynne K, Baker NB, Hunter J, Carthy T, Booker E, London M, Deakin JF, Sahakian BJ, Robbins TW (1999). Dissociable deficits in the decision-making cognition of chronic amphetamine abusers, opiate abusers, patients with focal damage to prefrontal cortex, and tryptophan-depleted normal volunteers: evidence for monoaminergic mechanisms. Neuropsychopharmacol.

[b52] Schoenbaum G, Setlow B, Nugent SL, Saddoris MP, Gallagher M (2003). Lesions of orbitofrontal cortex and basolateral amygdala complex disrupt acquisition of odor-guided discriminations and reversals. Learn. Memory.

[b53] Schoenbaum G, Saddoris MP, Ramus SJ, Shaham Y, Setlow B (2004). Cocaine-experienced rats exhibit learning deficits in a task sensitive to orbitofrontal cortex lesions. Eur. J. Neurosci.

[b54] Tsuchida A, Doll BB, Fellows KL (2010). Beyond reversal: a critical role for human orbitofrontal cortex in flexible learning from probabilistic feedback. J. Neurosci.

[b55] Vanderschuren LJ, Everitt BJ (2004). Drug seeking becomes compulsive after prolonged cocaine self-administration. Science.

[b56] Volkow ND, Fowler JS (2000). Addiction, a disease of compulsion and drive: involvement of the orbitofrontal cortex. Cereb. Cortex.

[b57] Volkow ND, Hitzemann R, Wang GJ, Fowler JS, Wolf AP, Dewey SL, Handlesman L (1992). Long-term frontal brain metabolic changes in cocaine abusers. Synapse.

[b58] Waldorf D, Reinarman C, Murphy S (1991). Cocaine Changes: The Experience of Using and Quitting.

[b59] Weissenborn R, Robbins TW, Everitt BJ (1997). Effects of medial prefrontal or anterior cingulate cortex lesions on responding for cocaine under fixed-ratio and second-order schedules of reinforcement in rats. Psychopharmacology.

[b60] Wilson JM (1996). Striatal dopamine nerve terminal markers in human, chronic methamphetamine users. Nat. Med.

[b61] Winstanley CA, Theobald DE, Cardinal RN, Robbins TW (2004). Contrasting roles of basolateral amygdala and orbitofrontal cortex in impulsive choice. J. Neurosci.

[b62] Xue Y, Steketee JD, Sun W (2012). Inactivation of the central nucleus of the amygdala reduces the effect of punishment on cocaine self-administration in rats. Eur. J. Neurosci.

[b63] Zapata A, Minney VL, Shippenberg TS (2010). Shift from goal-directed to habitual cocaine seeking after prolonged experience in rats. J. Neurosci.

