# Supplementary figures and images for: Differential roles of the prefrontal cortical subregions and basolateral amygdala in compulsive cocaine seeking and relapse after voluntary abstinence in rats
Source: Eur J Neurosci. 2013 Jul 1;38(7):3018–26. doi: 10.1111/ejn.12289 (PMC3910160; doi:10.1111/ejn.12289)

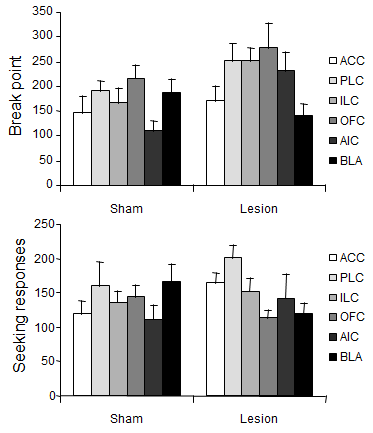

Supplement: Fig S1 — Break point under the progressive ratio schedule and seeking responses during the non-reinforced seeking probe test in which only the seeking lever was presented in rats with lesions to the anterior cingulate cortex, prelimbic cortex, infralimbic cortex, orbitofrontal cortex, anterior insular cortex and basolateral amygdala and their counterpart sham controls. [file ejn0038-3018-sd1.tif]

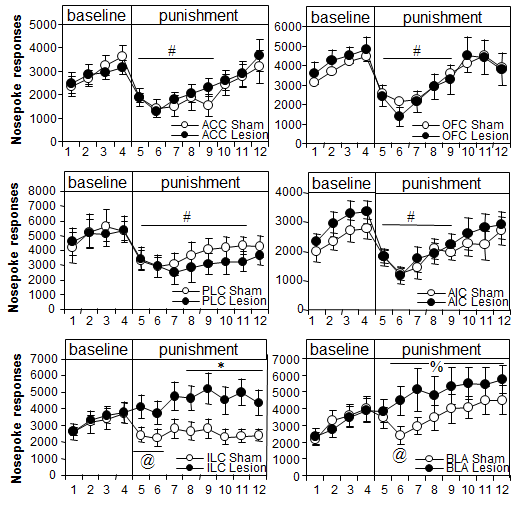

Supplement: Fig S2 — Number of nose poke responses over the 4 days before (baseline) or during the 8 days (punishment) of punishment of rats with lesions to the anterior cingulate cortex, prelimbic cortex, infralimbic cortex, orbitofrontal cortex, anterior insular cortex and basolateral amygdala and their counterpart sham controls. [file ejn0038-3018-sd2.tif]

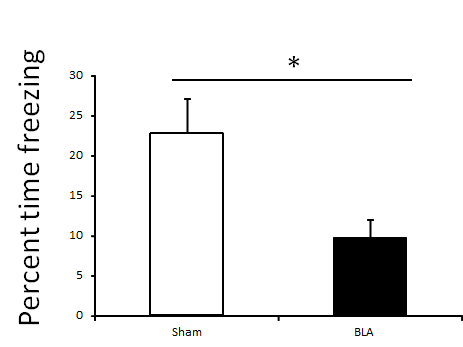

Supplement: Fig S3 — Percentage of time spent in freezing during intermittent presentation of a 1-min conditioned stimuli in rats with lesions to the basolateral amygdala and their counterpart sham controls. [file ejn0038-3018-sd3.tif]

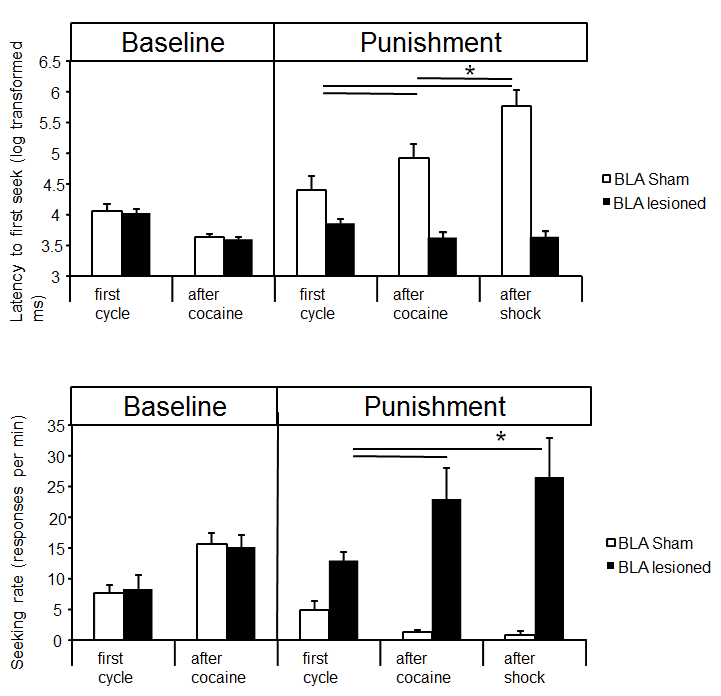

Supplement: Fig S4 — Latency to the first seeking response and seeking rate during baseline or during punishment either during the first cycle, or after a previous cocaine-reinforced cycle, or after a previously punished cycle, in rats with lesions to the BLA basolateral amygdala and their counterpart sham controls. [file ejn0038-3018-sd4.tif]
