# Supplementary material for: Differential roles of the prefrontal cortical subregions and basolateral amygdala in compulsive cocaine seeking and relapse after voluntary abstinence in rats
Source: Eur J Neurosci. 2013 Jul 1;38(7):3018–26. doi: 10.1111/ejn.12289 (PMC3910160; doi:10.1111/ejn.12289)
Supplement: Table S1 — Infusion coordinates for infusion (Chudasama et al., 2003; Ito et al., 2005; Boulougouris et al., 2007; Kesner & Gilbert, 2007). [file ejn0038-3018-sd5.docx]

|  | | Coordinates | | | | | Volume (μl) | | | Source | |  |
| --- | --- | --- | --- | --- | --- | --- | --- | --- | --- | --- | --- | --- |
| AP | | ML | | DV | |  | | |  | |
| ACC | + 3.2 | | ± .7 | | -1.9 | | 0.4 | | Chudasama *et al.,* 2003 | | | |
| + 2.7 | | ± .7 | | - 1.9 | | 0.4 | |
| + 2.2 | | ± .7 | | - 1.9 | | 0.4 | |
| PLC | + 4 | | ± .8 | | - 3.3 | | 0.4 | | Boulougouris *et al.,* 2007 | | | |
| + 2.7 | | ± .8 | | - 3.8 | | 0.4 | |
| ILC | + 3 | | ± .7 | | - 4.5 | | 0.4 | | Chudasama *et al.,* 2003 | | | |
| + 2.5 | | ± .7 | | - 4.5 | | 0.4 | |
| OFC | + 4.2 | | ± .8 | | - 3.4 | | 0.4 | | Modified from Chudasam et al., 2003 | | | |
| + 3.7 | | ± 1.8 | | - 3.6 | | 0.4 | |
| + 3.2 | | ± 2.8 | | - 4.4 | | 0.4 | |
| AIC | + 4.9 | | ± 2.6 | | - 3 | | 0.6 | | Kesner & Gilbert, 2007 | | | |
| + 3.9 | | ± 3.7 | | - 4.4 | | 0.6 | |
| + 2.9 | | ± 4.5 | | - 4.8 | | 0.6 | |
| BLA | - 2.3 | | ± 4.6 | | - 7.3 | | 0.4 | | Ito *et al.*, 2005 | | | |
| - 3.0 | | ± 4.6 | | - 7.3 | | 0.4 | |

Supplementary Table 1: Infusion coordinates for infusion. ACC (Chudasama *et al.,* 2003); PLC (Boulougouris *et al.,* 2007); ILC (Chudasama *et al.,* 2003); OFC (modified from Chudasama *et al.,* 2003); AIC (Kesner & Gilbert, 2007); BLA (Ito *et al.*, 2005).

**References**

Boulougouris, V., Dalley, J.W., Robbins, T.W. (2007) Effects of orbitofrontal, infralimbic and prelimbic cortical lesions on serial spatial reversal learning in the rat. *Behav*. *Brain Res*., **179**, 219-228.

Chudasama, Y., Passetti, F., Rhodes, S.E., Lopian, D., Desai, A. & Robbins, T.W. (2003) Dissociable aspects of performance on the 5-choice serial reaction time task following lesions of the dorsal anterior cingulate, infralimbic and orbitofrontal cortex in the rat: differential effects on selectivity, impulsivity and compulsivity. *Behav*. *Brain Res*., **146**, 105–119.

Ito, R., Everitt, B.J. & Robbins, T.W. (2005) The hippocampus and appetitive Pavlovian conditioning: effects of excitotoxic hippocampal lesions on conditioned locomotor activity and autoshaping. *Hippocampus*, **15**, 713–721.

Kesner, R.P. & Gilbert, P.E. (2007) The role of the agranular insular cortex in anticipation of reward contrast. *Neurobiol*. *Learn*. *Mem*., **88**, 82–86.
